# A case series of acute ischemic strokes with contralateral perfusion time delay on brain computed tomography

**DOI:** 10.1097/MD.0000000000033790

**Published:** 2023-05-12

**Authors:** Ji Min Lee, Yu Jeong Shin, Shin Byoung-Soo, Hyun Goo Kang

**Affiliations:** a Jeonbuk National University Medical School, Jeonju, South Korea; b Department of Neurology & Research Institute of Clinical Medicine of Jeonbuk National University – Biomedical Research Institute of Jeonbuk National University Hospital, Jeonju, South Korea.

**Keywords:** acute ischemic stroke, cerebral infarction, collateral circulation, perfusion, stenosis

## Abstract

Collateral circulation sustains cerebral perfusion in patients with arterial occlusion. Extensive arterial occlusion may redirect cerebral blood flow to compensate for insufficient perfusion. Cerebral artery occlusion can be observed in computed tomography perfusion imaging with increased mean transit time (MTT). However, in some cases, MTT delay occurs contralateral to the site of stenosis or occlusion. This delay cannot be explained simply by the collateral blood supply. Therefore, the authors considered the similarity of the perfusion delay observed at the normal site to that observed in subclavian steal syndrome.

**Case presentation:**

Three patients were reviewed: the first had severe stenosis in the left proximal internal carotid artery (ICA), and the second had left common carotid artery occlusion and diffusion restriction of the ICA-middle carotid artery border zone. The third patient had total occlusion of the left common carotid artery and right proximal ICA, with multifocal infarctions in the right frontal, occipital, left frontal, and parietal lobes. All 3 patients had a contralateral MTT delay on perfusion imaging.

**Conclusion:**

The site of stenosis or occlusion did not correlate with ipsilateral perfusion delay in these 3 cases. Based on the precedent relationship between infarction and perfusion delay, we developed 2 hypotheses to explain why perfusion decreases on the contralateral side of the occlusion or stenosis. However, this study was limited because we could not identify events, like volume loss or decreased blood pressure, before stroke development.

## 1. Introduction

Collateral circulation sustains cerebral perfusion in cases of arterial occlusion, thereby preventing cerebrovascular disease. The presence and development of collateral flow determine the degree of impairment in cerebral hemodynamics when arterial occlusion or stenosis occurs.^[1,2]^

Extensive arterial occlusion may redirect cerebral blood flow (CBF) to compensate for insufficient perfusion. A representative syndrome is the subclavian steal syndrome (SSS), which refers to retrograde flow in the vertebral artery resulting from substantial vertebrobasilar ischemia secondary to severe stenosis or occlusion of the proximal subclavian or innominate artery.^[3]^ When the common carotid artery (CCA) or innominate artery is obstructed, blood flows from the external carotid artery to the ipsilateral internal carotid artery (ICA). This collateral flow is called the carotid steal or external carotid artery-to-ICA steal phenomenon.^[4]^

Cerebral artery occlusion can be observed on computed tomography (CT) perfusion imaging. It is characterized by increased mean transit time (MTT), decreased CBF, and cerebral blood volume (CBV) in regions supplied by the artery.^[5]^ These findings suggest that the failure to develop collateral pathways induces decreased perfusion. However, we encountered cases where the vascular occlusion and CT perfusion findings were incongruent. Herein, we report these cases from the perspective of collateral circulation.

## 2. Case presentation

### 2.1. Case 1

A right-handed 48-year-old man was admitted for transient right-sided hemiparesis after drinking alcohol an hour before his presentation. The symptoms lasted approximately 2 minutes before resolution and were not accompanied by dysarthria. The patient had hypertension, chronic alcoholism, and a smoking history of 30 pack years. He did not have heart disease, hypercholesterolemia, diabetes, autoimmune disease, or angiopathies such as vasculitis. Neurological examination on admission revealed intact consciousness, no dysarthria or aphasia, no hemiparesis, and intact deep tendon reflexes in all extremities. Pathological reflexes were not observed. No abnormalities were observed in the blood, electrolytes, liver function, or renal function test results.

**Figure 1.**
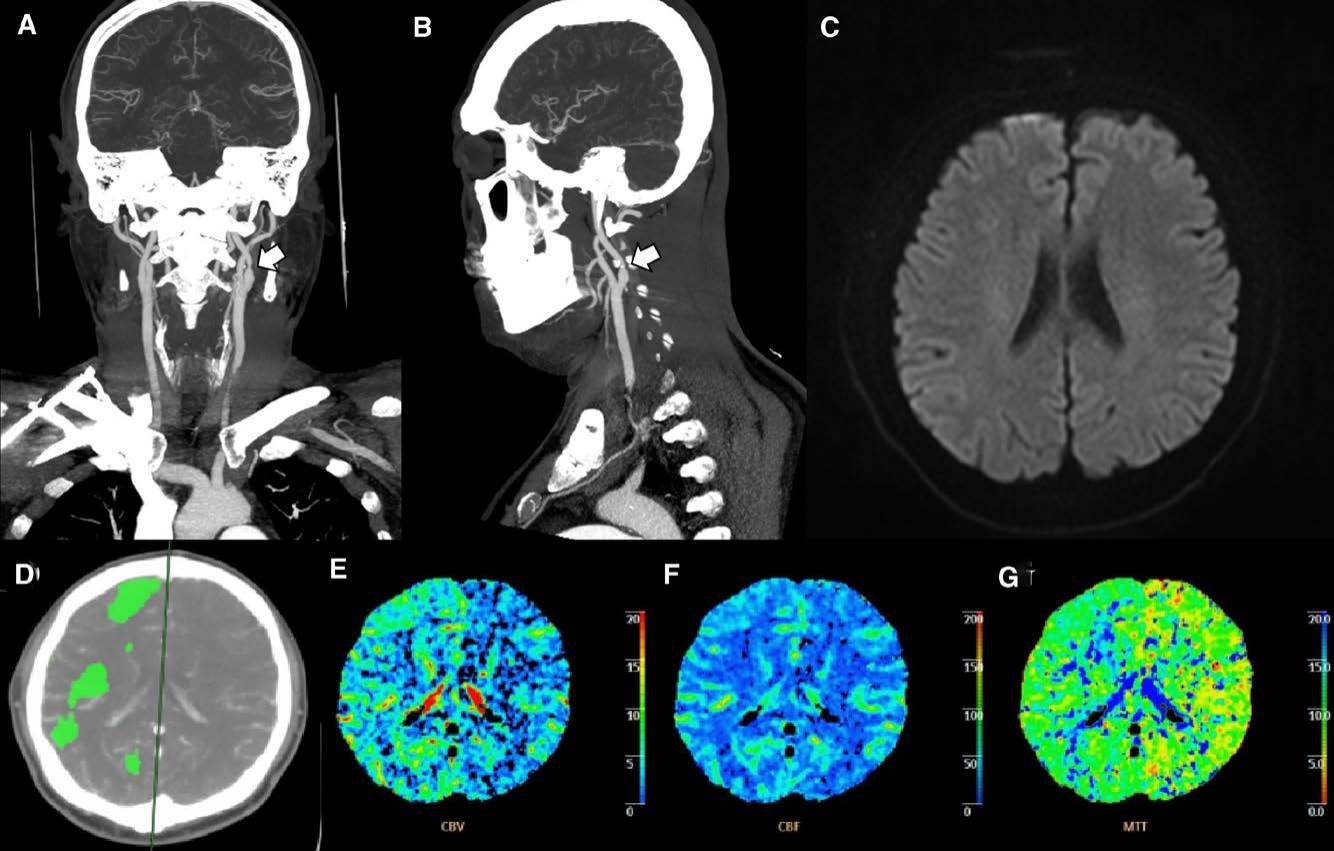
Transient right hemiparesis with left internal carotid artery (ICA) stenosis (Case 1). (A) Left ICA stenosis (arrow) in coronal computed tomography angiography (CTA) imaging. (B) Left ICA stenosis (arrow) in sagittal CTA imaging. (C) No restriction on diffusion-weighted magnetic resonance imaging. (D–G) Perfusion CTA images show similar cerebral blood flow on both sides but increased mean transit time and cerebral blood volume on the right hemisphere.

Similarly, non-contrast CT conducted at admission revealed no abnormalities. However, CT angiography (CTA) revealed severe stenosis of the left proximal ICA (Fig. 1A and B). The right carotid, middle cerebral, and vertebrobasilar arteries were unaffected, but there was an increase in MTT in the right hemisphere on CT perfusion imaging (Fig. 1D–G). No restriction was observed on diffusion-weighted imaging (DWI) or magnetic resonance imaging (MRI) (Fig. 1C).

### 2.2. Case 2

**Figure 2.**
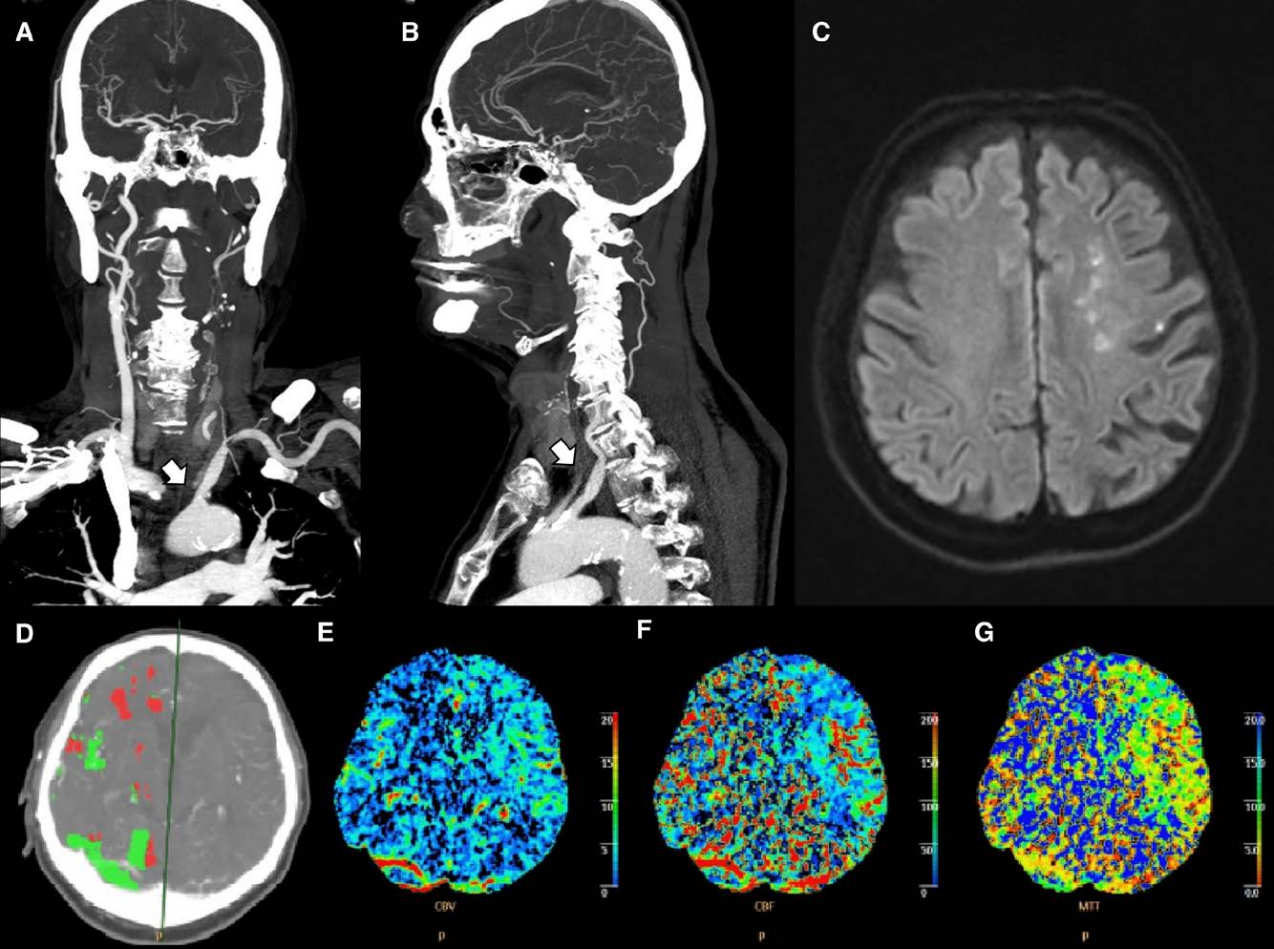
Left middle cerebral artery territory infarction with left CCA occlusion (Case 2). (A) Left common carotid artery (CCA) occlusion (arrow) in coronal computed tomography angiography (CTA) imaging. (B) Left CCA occlusion (arrow) in sagittal CTA imaging. (C) The diffusion restriction of the left internal carotid artery (ICA)-middle cerebral artery (MCA) internal border zone in (D–G) diffusion-weighted magnetic resonance imaging. Perfusion CTA images show increased mean transit time and decreased cerebral blood flow and blood volume on the right hemisphere. CCA = common carotid artery.

A 76-year-old man was admitted to our hospital with right-leg weakness that occurred on the day of admission. He had a history of transient right-leg weakness, lasting 1 to 2 minutes twice daily, starting 7 days before admission. The patient had a history of hypertension and diabetes and was taking aspirin as prophylaxis. He was alert, and there were no abnormal findings on cranial nerve examination. However, the muscle strength of the right upper and lower extremities decreased to Medical Research Council grade 4. There was no paresthesia, deep tendon reflexes were normal, and the Babinski reflex was not observed. His National Institutes of Health Stroke Scale score was 2. Although there were no abnormalities on non-contrast CT at admission, left CCA occlusion was observed on CTA (Fig. 2A and B). An increase in MTT was also observed in the right hemisphere on CT perfusion imaging (Fig. 2D–G), and diffusion restriction of the left ICA-middle carotid artery internal border zone was confirmed on MRI DWI (Fig. 2C).

### 2.3. Case 3

**Figure 3.**
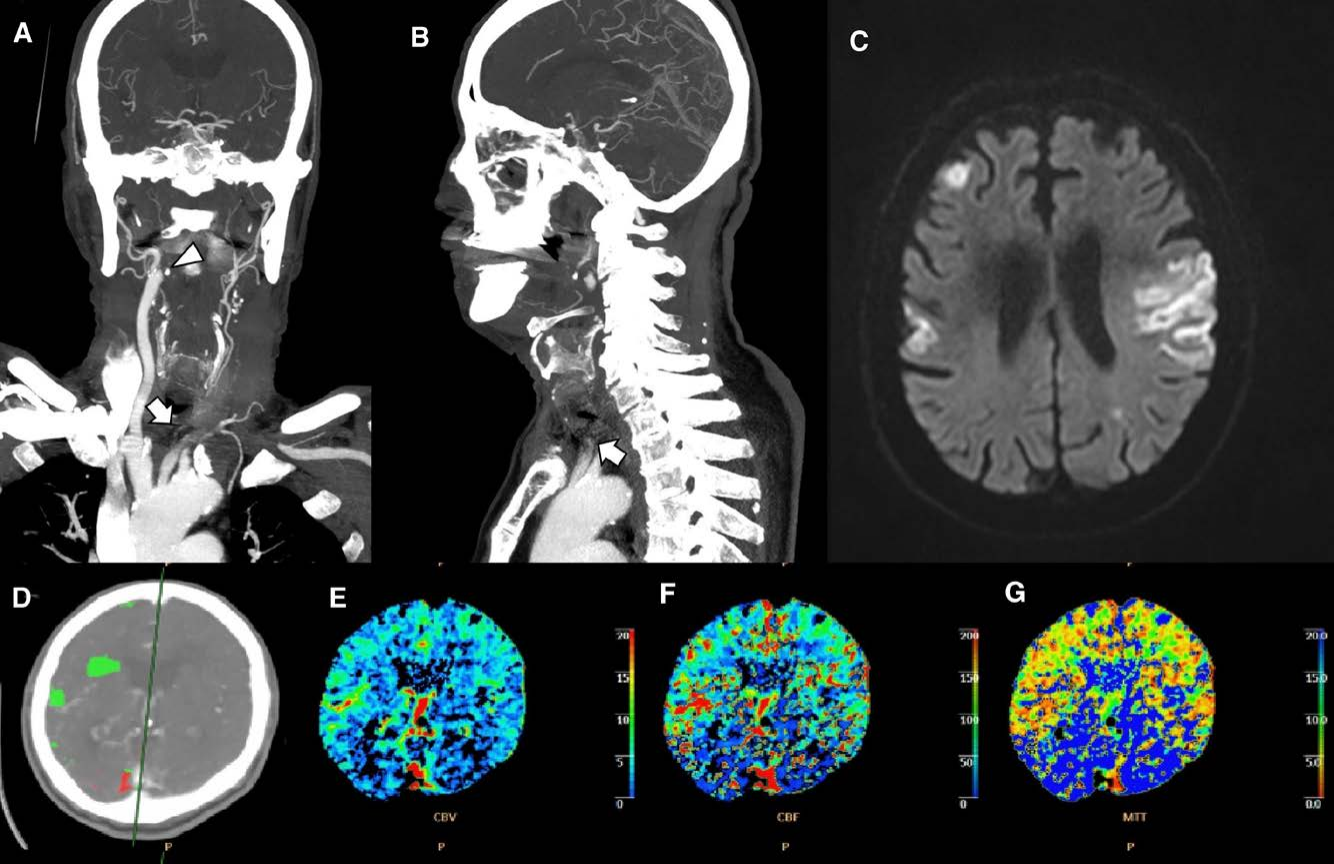
Left middle cerebral artery territory infarction with left CCA and right ICA occlusion (Case 3). (A) Left common carotid artery (CCA, arrow) and right proximal ICA occlusion (arrowhead) in coronal computed tomography angiography (CTA) imaging. (B) Left CCA occlusion (arrow) in sagittal CTA imaging. (C) There is multifocal infarction in the bilateral parietal and frontal lobes on (D–G) diffusion-weighted magnetic resonance imaging. Perfusion CTA images show increased mean transit time and decreased cerebral blood flow and cerebral blood volume in the posterior circulation. CCA = common carotid artery, ICA = internal carotid artery.

An 81-year-old right-handed man became unconscious 3 hours and 30 minutes before admission. He was transported to the hospital by ambulance. The patient had hypertension, but no heart disease, hypercholesterolemia, or diabetes. Neurological examination revealed that the patient was deeply drowsy. The patient had isochoric pupils (3 mm) with normal light reflexes, mild dysarthria, right facial palsy, and left eyeball deviation. The Medical Research Council grades of his left upper and lower extremities were 3 and 4, respectively, while those of the right upper and lower extremities were 2. Hypoesthesia was observed on the patient’s right side. Deep tendon reflexes were normal, and the Babinski reflex was not observed. His National Institutes of Health Stroke Scale score was 25. Although non-contrast CT conducted on admission showed no abnormal findings, CTA revealed total occlusion of the left CCA and the right proximal ICA (Fig. 3A and B). CT perfusion imaging confirmed increased MTT in the bilateral thalamus, cerebellum, brain stem, and all territories of the basilar artery (Fig. 3D–G). MRI DWI showed multifocal infarctions in the bilateral frontal and parietal lobes and left occipital area (Fig. 3C).

## 3. Discussion and conclusions

The cases reported in this study involved severe chronic artery stenosis or occlusion, with incongruent areas of reduced perfusion. No preexisting arterial stenoses or infarctions were observed in the regions with decreased perfusion. Perfusion can decrease due to ICA stenosis or occlusion, poor cardiac output secondary to arrhythmias (e.g., atrial fibrillation), and intracranial vessel occlusion.^[6,7]^ Perfusion CT in patients with ischemic stroke typically shows decreased CBF and CBV and increased MTT in the infarction core. However, mismatches (increased MTT and normal CBV) can also occur in the penumbra. Therefore, we focused on MTT rather than CBV as a more sensitive marker of perfusion delay.^[8]^

The left proximal ICA stenosis presented as transient right-sided hemiparesis in case 1. Thus, perfusion delay was expected in the left hemisphere. However, MTT was delayed in the right hemisphere. In Case 2, the subject had right lower extremity monoparesis. He had left CCA occlusion on CTA and left ICA-middle carotid artery border zone infarction on brain MRI.

Nevertheless, an MTT delay was observed on the right side of the perfusion image. We speculate that the delay in perfusion was caused by the diversion of blood from the contralateral or normal hemisphere to the site of occlusion owing to the increased perfusion requirement on the ipsilateral side. The redirection of blood flow may be maintained through collateral pathways in the contralateral hemisphere.

Case 3 differs from the previous 2 cases. In case 3, total occlusion of the left CCA and the right proximal ICA was confirmed. Thus, the right vertebral artery was relatively well-developed, and both anterior circulations were occluded. However, a perfusion (MTT) delay was found in the bilateral thalamus, cerebellum, and brain stem, which are supplied by posterior circulation. These findings indicate that posterior circulation forms collateral blood supply to the anterior circulation. Although occlusions occurred in the anterior circulation (i.e., CCA and ICA), they were considered to have diverted from the posterior circulation, resulting in relatively decreased perfusion in the posterior regions.

Similarities were found between SSS and the diseases encountered in these cases. On the contralateral side of the occlusion or stenosis, decreased blood flow was observed in SSS. Decreased perfusion was also observed in our cases. Several causes may be attributed to decreased perfusion, including volume loss due to diarrhea or bleeding and a sudden drop in blood pressure or autonomic dysfunction. Based on the precedent relationship between infarction and perfusion delay, we developed 2 hypotheses to explain why perfusion decreases on the contralateral side of the occlusion or stenosis. The first hypothesis is that collateral blood is excessively supplied because of increased oxygen demand secondary to infarction or ischemia at the occlusion site – blood flow diversion results in decreased contralateral side perfusion. The second hypothesis speculates that a certain event first decreases the perfusion at the normal site, leading to cerebral infarction and transient ischemic attack at the occlusion site receiving blood from the normal site. The occlusion site did not exhibit a decrease in MTT because of compensatory mechanisms that supplied blood through the collateral vessels. However, perfusion delay may occur in the normal hemisphere, which has low adaptability.

This study was limited because we could not check for the occurrence of events before the onset of stroke or its symptoms. We did not observe unusual findings in the patient’s medical history or physical condition. We could also not check the function of the arteries supplying collateral flow, including the Circle of Willis, by using medical imaging. Nevertheless, these cases were meaningful because we observed a phenomenon similar to Steal syndrome, which decreased perfusion contralateral to the occlusion site.

Neither hypothesis was predominant. Both can help diagnose similar patients and predict their complications. For example, decreased perfusion contralateral to infarction. Therefore, it is necessary to consider the possibility of infarction or transient ischemic attack on the normal side. Further studies should be conducted to understand the temporal order of infarctions and contralateral decreased perfusion.

## Author contributions

**Conceptualization:** Hyun Goo Kang.

**Data curation:** Ji Min Lee, Yu Jeong Shin, Shin Byoung-Soo.

**Formal analysis:** Ji Min Lee, Yu Jeong Shin, Shin Byoung-Soo, Hyun Goo Kang.

**Methodology:** Hyun Goo Kang.

**Software:** Ji Min Lee, Yu Jeong Shin.

**Supervision:** Shin Byoung-Soo, Hyun Goo Kang.

**Validation:** Shin Byoung-Soo.

**Visualization:** Yu Jeong Shin.

**Writing – original draft:** Ji Min Lee, Yu Jeong Shin, Hyun Goo Kang.

**Writing – review & editing:** Hyun Goo Kang.
